# Cyto-IL-15 synergizes with the STING agonist ADU-S100 to eliminate prostate tumors and confer durable immunity in mouse models

**DOI:** 10.3389/fimmu.2023.1196829

**Published:** 2023-07-03

**Authors:** Efthymia Papaevangelou, Ana M. Esteves, Prokar Dasgupta, Christine Galustian

**Affiliations:** ^1^ Peter Gorer Department of Immunobiology, School of Immunology and Microbial Sciences, King’s College London, Guy’s Hospital, London, United Kingdom; ^2^ Institute of Medical and Biomedical Education, St. George’s University of London, London, United Kingdom; ^3^ Urology Centre, Guy’s Hospital, London, United Kingdom

**Keywords:** immunotherapy, IL-15, STING agonist, prostate cancer, abscopal immunity

## Abstract

**Introduction:**

Prostate cancer is one of the most commonly diagnosed malignancies in men with high mortality rates. Despite the recent therapeutic advances, such as immunotherapies, survival of patients with advance disease remains significantly low. Blockade of immune checkpoints has led to low response rates in these patients probably due to the immunosuppressive microenvironment and low mutation burden of prostate tumors. Combination of multiple immunotherapeutic regimes has also been unsatisfactory due to augmented adverse effects. To activate multiple immune-stimulatory pathways in the hostile prostate cancer microenvironment, we used a combination of cytotopically modified interleukin-15 (cyto-IL-15) with the stimulator of interferon genes (STING) agonist, ADU-S100.

**Methods:**

To determine whether this combination regime could lead to both local and systemic anti-tumor effects, intratumoral administration of these agents was used in murine models of prostate cancer. Tumor growth and mouse survival were monitored, and ex vivo analyses, and RNA sequencing were performed on the tumors.

**Results:**

Intratumorally injected ADU-S100 and cyto-IL-15 synergized to eliminate tumors in 58-67% of mice with unilateral tumors and promoted abscopal immunity in 50% of mice with bilateral tumors treated only at one side. Moreover, this combination regime offered immunoprotection against tumor rechallenge in 83% of cured mice. The efficacy of the combination treatment was associated with a strong innate and adaptive immune activation and induction of apoptotic and necrotic cell death. Cytokines, including type I and II interferons, and cytokine signalling pathways were activated, NK and T cell mediated cytotoxicity was increased, and B cells were activated both locally and systemically. While ADU-S100 led to an ulcerative pathology at the injection site, no other adverse effects were observed.

**Discussion:**

Localised administration of a STING agonist together with cyto-IL-15 can confer significant systemic benefits and long-lasting immunity against prostate tumors while reducing immune related toxicities.

## Introduction

Prostate cancer is the most commonly diagnosed cancer in men and one of the leading causes of cancer-related male mortality worldwide (1). Over the last two decades, although the death rate has decreased due to significant advances in prostate cancer therapies, incidence rates have increased (2). Moreover, patients with advanced disease have a 5-year overall survival of only 30% (3).

Immunotherapies for prostate cancer have gained scientific interest due to the success of these treatments in other cancer types. The first FDA-approved immunotherapy for metastatic, castration-resistant prostate cancer (mCRPC) was Sipuleucel-T (Provenge), an autologous dendritic cell vaccine offering immunization against prostatic acid phosphatase. However, Sipuleucel-T has a high cost and results in a tangible survival benefit of four months (4). Other immunotherapeutic approaches used in prostate cancer clinical trials include checkpoint inhibitory antibodies against cytotoxic T-lymphocyte antigen 4 (CTLA-4), such as ipilimumab, and programmed cell death protein 1 (PD-1), such as nivolumab or pembrolizumab. Despite durable responses of these checkpoint inhibitors in other cancers, their efficacy against prostate cancer, both when used as monotherapies or combined, is minimal and only benefits a small cohort of patients (5–8). Pembrolizumab is the only FDA-approved checkpoint inhibitor for treating prostate cancer, but only for patients with high microsatellite instability and/or mutations in mismatch repair genes in the tumor (9). The low response of prostate cancer to immunotherapies has been attributed to the low somatic mutation burden, and the immunosuppressive prostate tumor microenvironment, which result in low number of neoantigens and poor infiltration of cytotoxic T cells, indicative of an immunologically “cold” non-T-cell inflamed tumor (10). Hence, there is an urgent need for identifying alternative immunotherapy combinations for treating prostate cancer that will holistically boost the immune response without dependency on neoantigens and pre-existing anti-tumor immunity. Such molecules include recombinant cytokines such as interleukin-15 (IL-15), and activators of the stimulator of interferon genes (STING).

IL-15 is a 14 kDa protein expressed by many immune cells, including macrophages, dendritic cells (DCs) and monocytes and it is known to stimulate the proliferation and activation of T cells and natural killer (NK) cells and their recruitment inside tumors (11). The anti-tumor potential of recombinant IL-15 has been demonstrated in murine tumors including prostate cancer (12, 13). Clinical trials have been conducted or are currently underway using IL-15 as monotherapy or in combination with ipilimumab and nivolumab in patients with advanced solid tumors rendering IL-15 a promising anti-cancer immunotherapy (14–16).

Another immunotherapeutic target is STING, an adaptor protein for the cytosolic DNA-sensing pathway cyclic GMP-AMP synthase (cGAS)-STING found in the endoplasmic reticulum. The cGAS-STING pathway activates the host immune response against tumors by inducing the production of pro-inflammatory cytokines including type I interferons (IFNs) (17). Tumor-derived cytosolic DNA is converted by c-GAS to cyclic di-nucleotides (CDNs), which bind to STING and activate interferon regulatory factors, such as IRF-3, leading to type I IFN production (18). Type I IFNs exert anti-tumor responses through a variety of effects on immune cells, such as promoting DC differentiation and maturation, and enhancing NK cell cytotoxicity (19). ADU-S100 is a synthetic CDN that has shown anti-tumor immunity in murine models when injected intratumorally (20, 21). Clinical trials in patients with advanced solid tumors or lymphomas have been conducted using ADU-S100 alone or in combination with checkpoint inhibitors. Moreover, a vast range of STING agonists are currently being developed in the preclinical or clinical stage (22).

The clinical responses to IL-15 or ADU-S100 monotherapies have not been as promising as in preclinical studies mainly due to adverse effects and dose-limiting toxicities. In the case of IL-15, despite the profound increase in circulating NK and CD8^+^ T cells, stable disease was the best response (16). In patients treated with ADU-S100, only partial responses were observed (22). Thus, we hypothesized that combination of a STING agonist, such as ADU-S100, with IL-15 could potentially increase the potency of both treatments by producing a synergistic effect, resulting in reduction of the required drug concentrations, and therefore reducing toxicities. IL-15 can stimulate NK and T cell proliferation and activation, and lead to type II interferon upregulation (i.e., IFN-γ), while the STING agonist can exert its effects through type I interferons (i.e., IFN-α and IFN-β). We previously showed that combination of IL-15 with an ADU-S100 analog in an *in vitro* prostate cancer-lymphocyte co-culture model increased NK cells cytotoxicity and led to notable cancer cell killing (23).

In this study, to identify prostate cancer treatments that are not only curative but also generate long-term immunoprotective responses, the potential of combining ADU-S100 with the membrane-localizing cyto-IL-15 was investigated *in vivo*. Their immunotherapeutic efficacy was explored in syngeneic and humanized murine models of prostate cancer, and systemic abscopal and long-lasting immunity were also examined. To reduce systemic adverse effects, both treatments were administered intratumorally. Mouse survival and tumor responses were monitored, and treatment effects were characterized using histopathology, cytokine analysis and RNA sequencing on tumor extracts.

## Materials and methods

### Cell lines

Transgenic adenocarcinoma of the mouse prostate (TRAMP)-C1 and TRAMP-C2 cells, obtained from American Type Culture Collection (ATCC, LGC Standards, Teddington, UK), were maintained in Dulbecco’s Modified Eagle’s culture medium (DMEM) supplemented with 2 mM L-glutamine, 1% antibiotic antimycotic solution, 0.2% gentamicin, 5 µg/mL insulin, 0.01 nM dihydrotestosterone (all from Sigma-Aldrich, Merck, Dorset, UK), 5% fetal bovine serum (FBS) (Life Technologies, Paisley, UK) and 5% NuSerum IV culture supplement (ThermoFisher Scientific, Dartford, UK). PC3 (human metastatic prostate epithelial carcinoma) cells, obtained from ATCC, were cultured in RPMI 1640 medium (Sigma-Aldrich) supplemented with 2 mM L-glutamine, 1% antibiotic antimycotic solution, 0.2% gentamicin, and 10% FBS. All cells were kept in a humidified atmosphere with 5% CO_2_ at 37°C and were negative for mycoplasma infection, which was tested frequently using LookOut Mycoplasma PCR (Sigma-Aldrich). Cell lines were used within 2 years from the date of purchase.

### Treatments

The STING-activating cyclic dinucleotide agonist ADU-S100 (MIW815; ML RR-S2 CDA) (MedChemExpress, Cambridge Bioscience Limited, Cambridge, UK) was diluted in HBSS (Hank’s balanced salt solution, vehicle) and was used at 50 µg (in 50µl) doses. IL-15 and cytotopically modified (cyto-IL-15) were produced in our laboratory as previously described (24), diluted in PBS and were used at 10 µg doses. Cyto-IL-15 is a version of IL-15 conjugated with a bis-myristoylated peptide to allow anchoring of IL-15 to cell membranes, which is in addition to IL-15 receptors binding. Currently a patent has been filled (P71020GB: KCL ref. 501/3048) for the combination of cyto-IL-15 with STING agonists in cancer treatment.

### Animals and tumors

Animal studies were performed in accordance with the UK Home Office Animals (Scientific Procedures) Act 1986 Animal and were reviewed and approved by the Animal Welfare Ethical Review Body (AWERB) Committee of King’s College London and by the Home Office, UK under Project Licence Number (PPL) P731DA7F1. Mice and tumors were monitored 2-3 times weekly for weight loss, hunched posture, discomfort, and development of rashes. The tumor length (L), width (W) and depth (D) were measured using calipers and the volume was calculated using the ellipsoid shape formula: (π/6) x L x W x D.

#### Single flank challenge

Male C57BL/6J mice, 6–8 weeks old (Charles River, Harlow, UK), were injected with 5 × 10^6^ TRAMP-C1 or C2 cells in 100 µl PBS subcutaneously into the right flank. When tumors reached approximately 100 mm^3^ in volume, mice were randomly divided into four treatment cohorts. Mice were injected intratumorally with 3 doses (every other day) of 50 µl HBSS or with 3 doses (every other day) of ADU-S100 (ADU) or 2 doses (day 0 and 4) of cyto-IL-15 or a combination of ADU (3 doses) and cyto-IL-15 (2 doses). Mice with TRAMP-C2 tumors were allocated to an additional cohort treated with 2 doses (day 0 and 4) of IL-15 and ADU (3 doses). The survival endpoint was when tumors reached a maximum diameter of 15 mm.

#### Rechallenge

Mice with TRAMP-C2 tumors (right flank) that were cured after treatment with ADU and cyto-IL-15 combination were rechallenged in the distal flank (left) with TRAMP-C2 (5 × 10^6^) cells. Rechallenge was performed 26-40 days after the original treated tumor complete regressed. Naïve mice of the same age were challenged on the left flank with TRAMP-C2 cells to be used as controls. Mice were culled 60 days after the rechallenge.

#### Tissue collection

Mice with TRAMP-C2 tumors of approximately 200 mm^3^ were randomly divided into four cohorts and treated as described above. Mice were culled at day 6 after treatment initiation to allow for tissue collection. Tumors were divided in parts and snap frozen. Spleens were used for single cell isolation. Blood was collected *via* cardiac puncture and mixed with 10% EDTA pH 8.0. Blood was centrifuge for 15 min at 2,000 x g at 4°C. The plasma (supernatant) was collected and stored frozen at -80°C until future use.

#### Bilateral flank challenge

Mice injected with TRAMP-C2 cells in the right flank were also injected with TRAMP-C2 cells (5 × 10^6^) in the distal left flank 2 weeks after the initial injection. When the initial (right flank) tumors reached approximately 50 mm^3^ in volume, mice were randomly divided into the four treatment cohorts and treated intratumorally only in the right flank as described above. The survival endpoint was when the maximum diameter of both right and left tumors reached a total of 15 mm.

#### Single flank challenge in humanized mice

HuNOG-EXL (HSCCB-13395-M, NOD.Cg-Prkdc Il2rgtm1Tg(SV40/HTLV-IL3,CSF2)10-7Jic/JicTac) male mice engrafted with human umbilical cord blood-derived CD34^+^ hematopoietic stem cells (HSCs) and with a human leukocyte reconstitution of ≥25% humanCD45^+^ cells in their blood at 10 weeks post engraftment were purchased from Taconic Biosciences (New York, US). Mice were injected with 10^7^ PC3 cells in 100 µl PBS subcutaneously into the right flank. Mice with ~100mm^3^ tumors were treated with HBSS or combination of ADU and cyto-IL-15 as described above. The survival endpoint was when tumors reached a maximum diameter of 15 mm or 50 days post tumor challenge if tumors completely regressed (<20 mm^3^).

### Cytokine bead array

TRAMP-C2 tumors were dissociated in PBS containing Complete Mini protease inhibitor tablets (Roche Diagnostics, West Sussex, UK) using a gentleMACS Dissociator (Miltenyi Biotec Ltd., Surrey, UK) according to the manufacturer’s instructions. Tumor lysates were kept at −80°C prior to analysis. Levels of IFN-γ, CXCL1 (KC), TNF-α, CCL2 (MCP-1), IL-12, CCL5 (RANTES), IL-1β, CXCL10 (IP-10), GM-CSF, IL-10, IFN-β, IFN-α and IL-6 were measured in tumor lysates and blood plasma using a LEGENDplex mouse anti-virus response panel kit (BioLegend, London, UK) following the manufacturer’s instructions. Data were acquired using a BD LSRFortessa cell analyzer (BD Biosciences, Wokingham, UK) and analyzed using VigeneTech software provided with the kit. All samples were measured in technical duplicates and biological replicates (*n* = 6). For tumor lysates, values were normalized to protein concentration of tumors, determined using a Pierce BCA protein assay Kit (ThermoFisher Scientific) according to manufacturer’s instructions. (IFN: interferon, TNF: tumor necrosis factor).

### Enzyme-linked immunosorbent assay (ELISA)

IL-15 receptor alpha (IL-15Rα) levels in tumor lysates were determined using a mouse IL-15Rα DuoSet ELISA (Bio-techne, R&D Systems, Abingdon, UK) according to manufacturer’s instructions, and values were normalized to tumor protein concentration.

### Histology and immunofluorescence

Frozen tumor sections (8 µm thick) were cut axially from two regions for each tumor, one in the center of the tumor and one 1 mm apart. To assess apoptosis, acetone-fixed sections were stained with a rabbit polyclonal anti‐cleaved caspase‐3 (CC3) antibody [AB3623] (1/200, Sigma-Aldrich) and Alexa Fluor 546 goat anti‐rabbit secondary antibody (2/1,000, ThermoFisher). Non‐immune‐specific rabbit IgG in the same concentrations as the anti‐CC3 antibody, was used as a negative isotype control. Staining was visualized under an Eclipse Ni-E Nikon fluorescent microscope using a Nikon DS-Fi3 camera (Nikon Instruments, Surrey, UK). For each tumor (*n* = 6 per treatment cohort), images were acquired from five randomly selected areas for each of the two tumor sections.

To assess the degree of necrosis, sections were stained with hematoxylin and eosin (H&E) and images were acquired using a bright-field Hamamatsu NanoZoomer 2.0RS digital slide scanner (Hamamatsu Photonics, Hamamatsu City, Japan). Tumor necrotic areas and fluorescent areas for CC3 were defined and analyzed blinded using ImageJ2 software (25) as previously described (26). Necrosis was expressed as a percentage of the whole tumor section area, while fluorescent staining as a percentage of the total image area.

### Flow cytometric analysis

Spleens were harvested from mice 6 days after treatment initiation and mechanically disrupted, passed through a 40 μm cell strainer, and rinsed with PBS to remove debris. Red blood cells were lysed using RBC lysis buffer. The isolated splenocytes were kept in -80°C until further use.

Splenocytes were thawed and stimulated with 1 μg/ml ionomycin and 20 ng/ml phorbol 12-myristate 13-acetate (PMA) (both from Sigma-Aldrich) for 4 h at 37°C. GolgiPlug protein transport inhibitor (containing Brefeldin A) (BD Biosciences) was also used to prevent intracellular marker release. Cells were collected, washed with PBS and stained for flow cytometry analysis. TruStain FcX™ PLUS (Biolegend) was used to block Fc receptors and a Cytofix/Cytoperm Plus Kit (BD Biosciences) was used according to manufacturer’s instructions for intracellular staining. The antibodies used are listed in **Table S1** (**Supplementary File 1**). Labelled cells were analyzed on the BD LSRFortessa cell analyser and data analysis was carried out using FlowJo version 10. The gating strategy for each immune population is shown in **Table S2** and **Figure S1** (**Supplementary File 1**).

### RNA sequencing

TRAMP-C2 tumors (collected at day 6 post-treatment) were used for RNA sequencing. Tumors were dissociated using a gentleMACS Dissociator according to manufacturer’s instructions and RNA was extracted using an RNeasy Mini Kit (Qiagen, Manchester, UK). RNA library preparations, sequencing reactions and bioinformatics analyses were conducted at Genewiz (Azenta Life Sciences, Leipzig, Germany) as described in Supplementary Methods (**Supplementary File 1**).

### Statistical analysis

Data were analyzed using GraphPad Prism 8 (GraphPad Software, La Jolla, CA). Statistical significance of differences was determined by one-way ANOVA with the appropriate multiple comparisons post-tests, with a 5% level of significance. Results are presented as mean ±1 standard error of the mean (SEM). Synergy calculations for ADU-S100 and cyto-IL-15 were performed with the combination index (CI) defining synergism (CI < 1), addition (CI = 1) or antagonism (CI > 1) (27, 28). For the RNA sequencing, the Wald test was used to generate p-values and Log2 fold changes. Genes with adjusted p-values <0.05 and absolute log2 fold changes >1 were defined as differentially expressed genes (DEGs) for each comparison.

## Results

### Cyto-IL-15 synergizes with the STING agonist ADU-S100 to clear prostate tumors in mice

**Figure 1:**
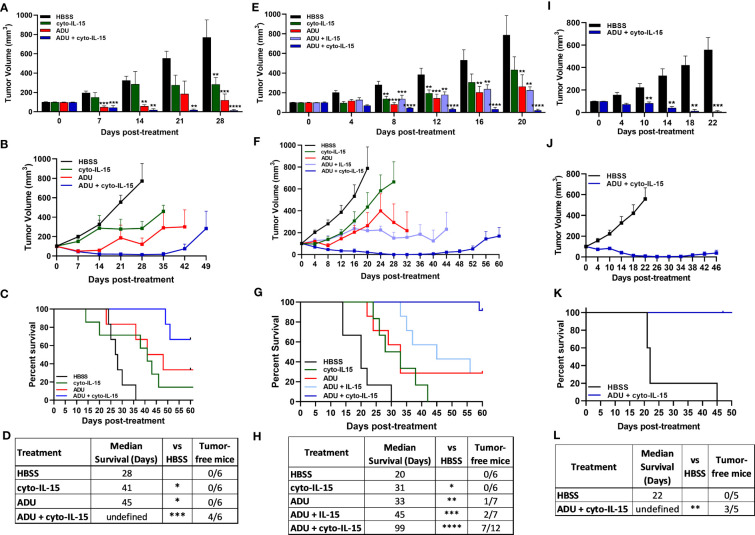
Combination of ADU-S100 with cyto-IL-15 leads to complete regression of subcutaneous prostate tumors in mice. **(A-D)** Mice with TRAMP-C1 tumors (right flank) treated intratumorally with HBSS, cyto-IL-15, ADU or combination of ADU with cyto-IL-15. **(A)** Tumor volumes up to day 28 after treatment (when most mice were still alive), and **(B)** tumor growth curves. Data are means + 1 SEM for *n* = 6 tumors per group and comparisons are relative to vehicle. **(C)** Survival curves of mice post-treatment and **(D)** table showing the median survival and the tumor-free mice of each group. **(E-H)** Mice with TRAMP-C2 tumors (right flank) treated intratumorally with HBSS, cyto-IL-15, ADU, combination of ADU with IL-15 or combination of ADU with cyto-IL-15. **(E)** Tumor volumes up to day 20 after treatment and **(F)** tumor growth curves. Data are means + 1 SEM for *n* = 6-12 tumors per group and comparisons are relative to vehicle. **(G)** Survival curves of mice post-treatment and **(H)** table showing the median survival and the tumor-free mice of each group. **(I-L)** Humanized mice with PC3 tumors treated intratumorally with HBSS or combination of ADU with cyto-IL-15. **(I)** Tumor volumes up to day 22 after treatment and **(J)** tumor growth curves. Data are means + 1 SEM for *n* =5 tumors per group and comparisons are relative to vehicle. **(K)** Survival curves of mice post-treatment, and **(L)** table showing the median survival and the tumor-free mice of each group. One-way ANOVA with Dunnett’s multiple comparisons post-test was used to compare tumor volumes and Log-rank (Mantel-Cox) test was used for comparisons of equality of two survival curves (*p < 0.05, **p <0.01, ***p <0.001, ****p <0.0001). Undefined survival means that more than 50% of the mice were still alive at the end of the study (60 days post-treatment if tumors did not regrow).

Intratumoral cyto-IL-15 can delay prostate tumor growth and increase survival, however it does not lead to complete regression of tumors even when combined with checkpoint inhibitors (12). To identify combination therapies that can clear prostate tumors, intratumoral cyto-IL-15 was combined with intratumoral STING activation using the STING agonist ADU-S100 (ADU). Mice with TRAMP-C1 syngeneic prostate tumors were treated with HBSS (vehicle), cyto-IL-15 or ADU monotherapies or combination of ADU and cyto-IL-15. Both monotherapies led to TRAMP-C1 tumor volume reduction (cyto-IL-15 by 63% and ADU by 84%) and increased survival (cyto-IL-15 to 41 and ADU to 45 days) compared with HBSS by day 28 post-treatment, which was the survival endpoint for the HBSS treated tumors (**Figures 1A–D**). However, only the combination treatment (cyto-IL-15 and ADU) led to complete tumor regression (98% volume reduction), had the greatest impact on survival (undefined, *p* < 0.001) and complete cured 4 out of 6 mice (67%). Cured tumor-free mice were defined as mice with complete tumor remission (tumor volume ≤20 mm^3^) and no tumor recurrence even after 60 days post-treatment initiation. Tumors on the non-cured mice started to regrow after day 35 post-treatment (**Figure 1B**). The effects of the combinatorial treatment were also examined in the TRAMP-C2 syngeneic prostate tumor model. Mice with TRAMP-C2 tumors were treated as those with TRAMP-C1 tumors and an additional cohort was treated with combination of ADU with IL-15 (not cytotopically modified) to examine whether the non-modified version of IL-15 has a similar effect in the combination regime. By day 20 (survival endpoint for mice with HBSS treated TRAMP-C2 tumors), ADU reduced tumor volume by 67% and combination of ADU with IL-15 by 71% compared with vehicle; both treatments increased survival to 33 and 45 days respectively (**Figures 1E–H**). Cyto-IL-15 significantly increased survival to 31 days and caused a small reduction in tumor volume (not statistically significant). However, similar to TRAMP-C1 tumors, only the combination treatment with cyto-IL-15 and ADU led to complete tumor regression (97% volume reduction), had the greatest impact on survival (99 days, *p* < 0.0001) and cured 7 out of 12 mice (58%) with TRAMP-C2 tumors. The remaining tumors started to regrow after day 44 post-treatment (**Figure 1F**). To examine whether the cyto-IL-15 and ADU combination treatment could also cure human prostate tumors, humanized mice with PC3 tumors were treated with HBSS or combination treatment. After 22 days of treatment (survival endpoint for mice with HBSS treated PC3 tumors), cyto-IL-15 combined with ADU led to complete tumor regression (98% volume reduction), significantly increased survival (undefined, *p <*0.01) compared to the HBSS treated cohort and cured 3 out of 5 mice (60%) (**Figures 1I–L**).

A synergy calculation was performed for cyto-IL-15 combined with ADU-S100 based on the volumes of TRAMP-C1 and C2 tumors at the survival endpoint of the HBSS control (days 28 and 20 post-treatment, respectively) (27, 28). The combination index was below one, more specifically 0.307 and 0.146 for TRAMP-C1 and TRAMP-C2 tumors respectively, indicating a strong synergistic effect between the two treatments (**Supplementary File 1**, **Table S3**).

Mice were monitored for adverse effects during the experimental period. Neither the monotherapies nor the combination treatment led to any significant weight loss (**Figure S2**, **Supplementary File 1**). However, intratumoral administration of ADU-100 alone or in combination with cyto-IL-15 caused an inflammatory reaction at the site of injection. Approximately a week after treatment initiation, ADU-S100 led to a skin ulceration in the C57BL/6J mice with bloody fluid that eventually turned into a scab and was resolved after 2 to 3 weeks. No ulcerative pathology was observed when cyto-IL-15 was administered alone. However, only 60% of the HuNOG-EXL (humanized) mice developed a scab after injection with the ADU-S100 plus cyto-IL-15 combination.

In addition, the spleen size of the mice was also monitored. Spleens harvested from mice treated with HBSS, cyto-IL-15 or cured mice after combination treatment had a normal size weighing 0.15 ± 0.04 g. However, splenomegaly was observed in mice treated with ADU-S100 or combination of ADU-S100 with cyto-IL-15 with progressing tumors with spleens weighing 0.51 ± 0.12 g.

### Intratumoral administration of ADU-S100 combined with cyto-IL-15 offers systemic antitumor immunity against distal tumors

IL-15 injected intravenously in nonhuman primates is known to cause adverse effects such as weight loss and neutropenia (29). In cancer patients, infusion of IL-15 leads to dose-limiting toxicities such as hypotension and thrombocytopenia (30). Hence, in the present study, intratumoral administration of the treatments was used to prevent systemic toxicities. To investigate whether intratumoral treatments not only prevent systemic adverse effects but can also generate systemic immune responses leading to immunoprotection and abscopal effects against distal tumors, two TRAMP-C2 tumor models were used, rechallenge and bilateral flank challenge, as described in the methods section.

**Figure 2:**
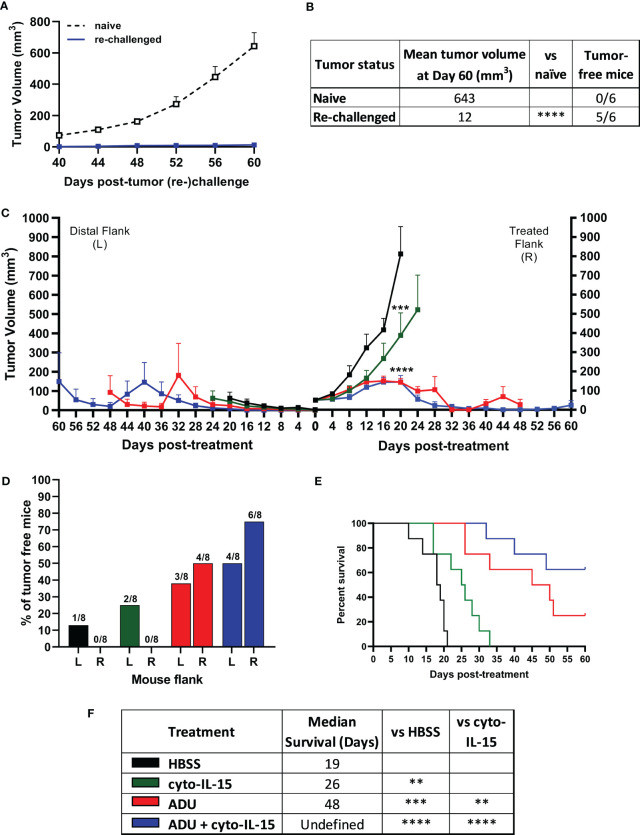
ADU-S100 combined with cyto-IL-15 generates systemic antitumor immunity in TRAMP-C2 subcutaneous prostate tumors in mice. **(A, B)** Mice with TRAMP-C2 tumors (right flank) that were cured after treatment with ADU and cyto-IL-15 combination were rechallenged in the distal flank (left) with TRAMP-C2 cells 26-40 days after the original treated tumor complete regressed. Naïve mice of the same age were challenged on the left flank with TRAMP-C2 cells to be used as controls. **(A)** Growth curves of distal tumors and **(B)** table showing the median tumor volume at Day 60 post-left tumor challenge and tumor-free mice per group. Data are means +1 SEM for *n* = 6 mice per group and tumor volumes were compared using unpaired t test (****p <0.0001). **(C-F)** Mice were challenged with TRAMP-C2 cells in the right flank and two weeks later they were challenged again with TRAMP-C2 cells in the distal (left) flank. When the initial right tumors were ~50 mm^3^ mice were treated intratumorally in the right flank only with HBSS, cyto-IL-15, ADU or combination of ADU with cyto-IL-15. **(C)** Tumor growth curves up to day 60 post-treatment, **(D)** percentage of tumor-free mice for both right and lefts tumors (numbers on top of the bars indicate actual mouse numbers), **(E)** survival curves of mice post-treatment, and **(F)** table showing the median survival of each treatment cohort. Data are means + 1 SEM for *n* = 8 mice per group and comparisons of equality of two survival curves were performed using Log-rank (Mantel-Cox) test (**p <0.01, ***p <0.001, ****p <0.0001).

Mice with TRAMP-C2 tumors treated with combination of cyto-IL-15 and ADU-S100 on the right flank and eventually cured of their tumor, were rechallenged in the distal (left flank) with TRAMP-C2 cells. Naïve mice, not previously injected with tumor cells, were also injected with TRAMP-C2 cells in the left flank to be used as controls. All the naïve mice (6/6) developed tumors on their left flanks approximately 40 days after the cell injection. On the contrary, 83% of the cured mice (5/6) did not develop tumors on their left flank even after 60 days of the rechallenge (**Figures 2A, B**). The tumor growth rate even for the one rechallenged mouse that developed a tumor was significantly slower compared with the tumor growth rate of the naïve mice. This indicates that the combination treatment can generate a long-lasting systemic immune response and protect against tumor recurrence.

Mice were challenged bilaterally with TRAMP-C2 cells and when they developed right flank tumors of approximately 50 mm^3^, they were treated intratumorally only in the right flank tumor with HBSS, cyto-IL-15, ADU-S100 or combination of cyto-IL-15 and ADU-S100 (as described in methods). Tumors treated with HBSS reached the survival endpoint (15 mm maximum diameter of right and left tumors together) at 20 days post-treatment, hence it was possible to observe the growth of the distal tumors in this group further. All treatments delayed the growth of the treated right-flank tumors compared with control treated tumors; a 63, 82 and 82% volume reduction was seen in cyto-IL-15, ADU-S100 and combination treated tumors, respectively (**Figure 2C**). Despite cyto-IL-15 alone slowing tumor growth, it did not lead to complete regression of any of the injected tumors (same as in the single flank challenge experiment), but it generated a small abscopal response in the distal (left flank) uninjected site with a 25% complete rejection. ADU-S100 monotherapy cleared 50% of the injected tumors and 38% of the distal ones. Combination treatment of ADU-S100 with cyto-IL-15 mediated rejection of 75% of injected tumors and elicited an abscopal response against 50% of distal uninjected tumors (**Figure 2D**). Cyto-IL-15, ADU-S100 or their combination significantly increased survival to 26, 48 or undefined days respectively, compared with 19 days in the HBSS group. Moreover, ADU-S100 alone or in combination increased survival significantly more compared with cyto-IL-15 alone. However, the combination had the highest overall impact on increasing mouse survival (**Figures 2E, F**). Thus, combination of cyto-IL-15 augments the curative abscopal immunity of ADU-S100 against TRAMP-C2 tumors with a complete bilateral tumor rejection in 50% of mice.

### ADU-S100 treatment alone or in combination with cyto-IL-15 induces severe cell death within six days of intratumoral administration

**Figure 3:**
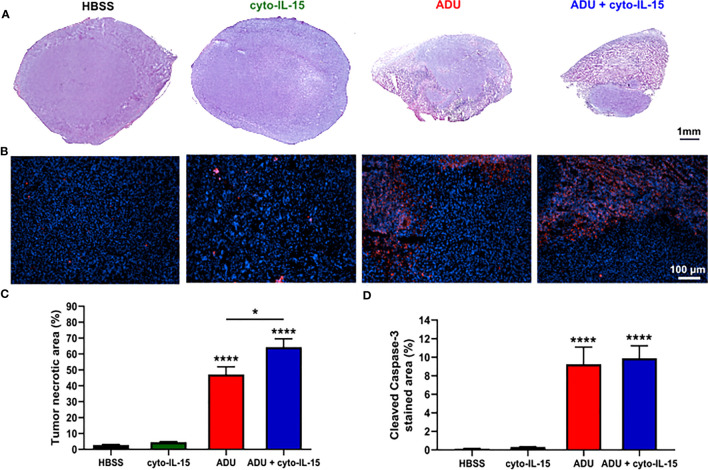
Histological assessment of treated TRAMP-C2 subcutaneous prostate tumors. **(A)** Composite images of H&E-stained sections indicating necrotic areas. **(B)** RGB images from tumor sections stained with the apoptotic marker cleaved caspase‐3 (CC3) detected using an Alexa‐546‐conjugated secondary antibody (red) and the nucleic marker DAPI (blue). **(C, D)** Quantification of **(C)** necrotic area and **(D)** cleaved caspase‐3 positive area. Results are means +1 SEM of 10 images per tumor for *n* = 6 per group. Comparisons are relative to vehicle unless otherwise indicated (*p <0.05, ****p <0.0001 one-way ANOVA with Tukey’s multiple comparisons post-test).

Combination of ADU-S100 and cyto-IL-15 can lead to complete regression of TRAMP-C2 tumors treated at 100 mm^3^ volume leaving none or minimal tumor tissue sample for analysis. Hence, mice with larger TRAMP-C2 tumors (200 mm^3^) were treated intratumorally at days 0, 2, and 4 and tumors were excised at day 6, as indicated in the methodology. Histological sections of the tumors after treatment with HBSS, cyto-IL-15, ADU-S100 or combination of cyto-IL-15 and ADU-S100 were stained with H&E and the early apoptotic marker, cleaved caspase-3 (**Figures 3A, B**). While cyto-IL-15 monotherapy was not sufficient to induce necrosis after 6 days of treatment, cyto-IL-15 in combination with ADU-S100 led to significant levels of necrosis (64%) compared with HBSS (3%). ADU-S100 monotherapy also caused high levels of necrosis (47%) compared with HBSS, but the effect was significantly lower compared with the combination (**Figure 3C**). Cleaved caspase-3 staining in the cyto-15 treated group was very low and similar to HBSS (0.3 and 0.1% respectively). However, caspase-3 activation was significantly increased after treatment with ADU-S100 alone or combined with cyto-IL-15 (9.2 and 9.9%, respectively) compared with HBSS (**Figure 3D**).

### Intratumoral injection of ADU-S100 with cyto-IL-15 activates type I IFNs and pro-infammatory cytokines and chemokines against tumors

**Figure 4:**
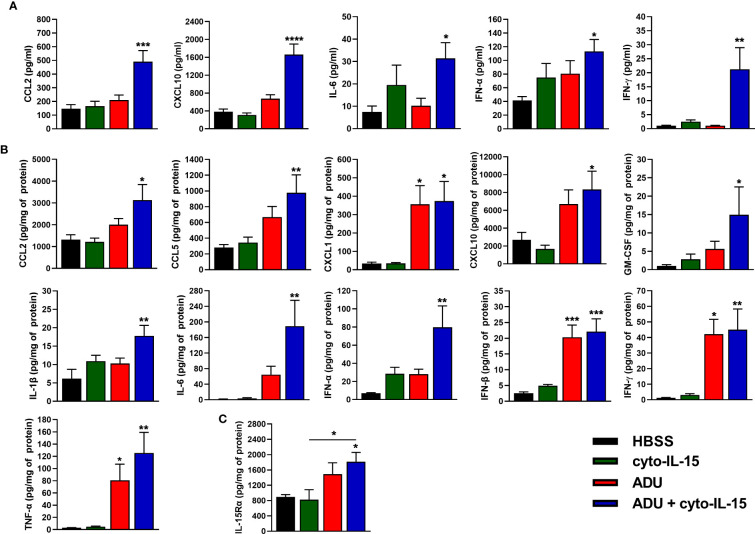
Cytokine activation in treated mice with TRAMP-C2 prostate tumors. **(A, B)** Concentration of cytokines in **(A)** blood plasma and **(B)** tumor lysates from mice treated with HBSS, cyto-IL-15, ADU or combination of ADU and cyto-IL-15 measured using a cytokine bead array. **(C)** Concentration of IL-15Rα in tumor lysates from the same mice measured with ELISA. Values for tumor lysates were normalized to tumor protein concentration. Results are means +1 SEM of duplicate measurements made for *n* = 6 mice per cohort. Comparisons are relative to vehicle unless otherwise indicated (*p <0.05, **p <0.01, ***p <0.001, ****p <0.0001 one-way ANOVA with Dunnett’s multiple comparisons post-test).

The effects of cyto-IL-15 and ADU-S100 on immune activation were investigated by measuring the release of cytokines in blood plasma and tumor lysates after 6 days of treatment using a cytokine bead array. In blood plasma of mice treated intratumorally with combination of ADU and cyto-IL-15, release of CCL2, CXCL10, IL-6, IFN-α and IFN-γ was significantly increased by 3.4- (*p <*0.001), 4.4- (*p <*0.0001), 4.2- (*p <*0.05), 2.7- (*p <*0.05) and 19.6-fold (*p <*0.01), respectively, compared with control (**Figure 4A**). Intratumoral monotherapy with ADU or cyto-IL-15 had no effect on cytokine release in blood plasma. In tumor lysates, combination treatment led to activation and release of most cytokines measured (**Figure 4B**). CCL2 (2.4-fold, *p <*0.05), CCL5 (3.5-fold, *p <*0.01), CXCL1 (11-fold, *p <*0.05), CXCL10 (3.1-fold, *p <*0.05), GM-CSF (14.5-fold, *p <*0.05), IL-1β (2.9-fold, *p <*0.01), IL-6 (121.5-fold, *p <*0.01), IFN-α (11.4-fold, *p <*0.01), IFN-β (8.8-fold, *p <*0.001), IFN-γ (36.8-fold, *p <*0.01) and TNF-α (43-fold, *p <*0.01) were all significantly increased compared with HBSS vehicle treatment. Moreover, ADU monotherapy significantly increased CXCL1 (10.5-fold, *p <*0.05), IFN-β (8.1-fold, *p <*0.001), IFN-γ (34.4-fold, *p <*0.05), and TNF-α (27.6-fold, *p <*0.05), compared with control, whereas cyto-IL-15 has no effect after 6 days of treatment. Release of IL-10 and IL-12(p70) in both tumor lysates and blood plasma was below detection level. For blood plasma, only cytokines with significant changes in expression are presented.

IL-15Rα expression was also measured in tumor lysates using ELISA. Combination treatment of ADU with cyto-IL-15 significantly increased IL-15Rα expression compared with HBSS (2-fold, *p <*0.05), and compared with cyto-IL-15 alone (2.2-fold, *p <*0.05) (**Figure 4C**).

### Intratumoral treatment with ADU-S100 and cyto-IL-15 leads to systemic immune cell activation and recruitment to the tumor site

To investigate the systemic effects of STING activation with ADU-S100 and IL-15 induction with cyto-IL-15, 15-color flow cytometry was used to characterize the immune cell composition of splenocytes. Splenocytes derived from spleens harvested from mice with TRAMP-C2 tumors treated intratumorally with HBSS, cyto-IL-15, ADU-S100 or combination of cyto-IL-15 and ADU-S100. Splenocytes were also stimulated *ex vivo* for 4 h with PMA and ionomycin to induce perforin and IFN-γ expression. Their extracellular release was blocked using GolgiPlug. The different immune subsets were defined as shown in **Table S2** and the gating strategy is shown in **Figure S1** (**Supplementary File 1**).

**Figure 5:**
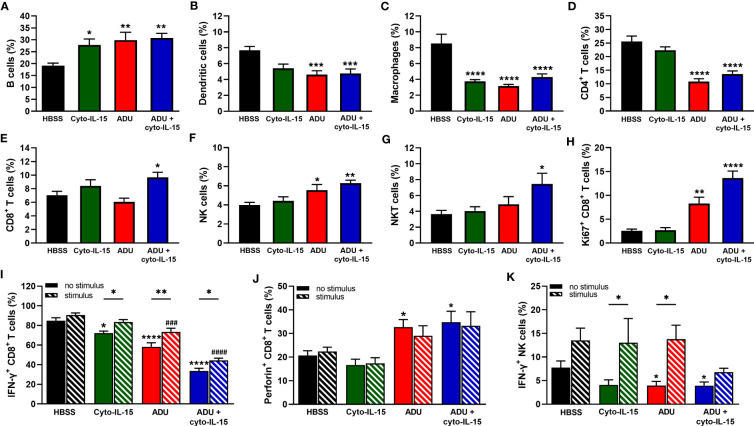
Analysis of immune cell composition of splenocytes in treated mice with TRAMP-C2 prostate tumors. Mice with TRAMP-C2 tumors (~200mm^3^) were treated intratumorally with HBSS, cyto-IL-15, ADU or combination of ADU and cyto-IL-15 and splenocytes were harvested after 6 days of treatment initiation to be analyzed using flow cytometry. **(A-H)** Frequencies of cell subsets within the CD45^+^ immune cell population: **(A)** B cells, **(B)** dendritic cells, **(C)** macrophages, **(D)** CD4^+^ T cells, **(E)** CD8^+^ T cells, **(F)** NK cells, and **(G)** NKT cells. **(H-K)** Frequencies of **(H)** Ki67^+^, **(I)** IFN-γ^+^ and **(J)** perforin^+^ CD8^+^ T cells, and **(K)** IFN-γ^+^ NK cells of splenocytes with or without PMA and ionomycin stimulation for 4 h. Results are means +1 SEM of measurements made for *n* = 6 mice per cohort. Comparisons are relative to control (* for HBSS and ^#^ for HBSS with stimulus) unless otherwise indicated (*p <0.05, **p <0.01, ***p <0.001, ****p <0.0001, ^###^ *p <*0.001, ^####^ *p <*0.0001 one-way ANOVA with Dunnett’s multiple comparisons post-test or Šidák’s for comparisons of stimulated versus non-stimulated samples).

As shown in **Figure 5A**, all treatments increased the frequency of B cells from 19% in the control group to approximately 30% (p <0.05 cyto-IL15; p <0.01 ADU-S100 and combination). The frequency of dendritic cells was reduced from 8% in the control to less than 6% in the ADU-S100 and combination treated mice (*p <*0.001) (**Figure 5B**). Macrophages constituted less than 9% of CD45^+^ cells in the control mice but this was significantly reduced to less than 4.5% in the treated mice (*p <*0.0001 with all treatments) (**Figure 5C**). A significant reduction was also observed in the frequency of CD4^+^ T cells from 22% in the control to less than 14% in the ADU-S100 and combination treated mice (*p <*0.0001) (**Figure 5D**). However, CD8+ T cells increased in the combination treated mice from >8% to 10% compared with control (*p <0.05) (**Figure 5E**). NK cells had higher frequency (>5.5%) in the ADU-S100 and combination treated mice compared with 4.4% in the control (p <0.05 ADU-S100; p <0.01 combination) (**Figure 5F**). NKT cells also increased from 4% in the control to 7.5% in the combination treated mice (*p <0.05) (**Figure 5G**). No changes were observed in the frequencies of myeloid derived suppressor cells (MDSCs) and regulatory T cells (Tregs) or in the M1/M2 composition of the macrophages between control and treated mice (**Figures S3A–C**, **Supplementary File 1**).

To investigate whether CD8^+^ T and NK cells were actively proliferating, the Ki67 proliferation marker was used. The frequencies of Ki67^+^ CD8^+^ T cells were higher in the ADU-S100 and combination treated groups increasing from 2.5 to >8% (p <0.01 ADU-S100; p <0.0001 combination) (**Figure 5H**). NK cells were more actively proliferating (>30%) compared with CD8^+^ T cells, but no significant differences were observed due to the treatments (**Figure S3D**, **Supplementary File 1**). The expression of IFN-γ and perforin were also investigated in the CD8^+^ T and NK cell populations. IFN-γ expression from CD8^+^ T cells was significantly reduced in the spleens after treatment from 85% in the control group to 72% in the cyto-IL-15 (p <0.05), 58% in the ADU-S100 (p <0.0001), and 34% in the combination group (p <0.0001). Despite IFN-γ expression significantly increasing after stimulation in the treated cohorts compared to their non-stimulated samples, it was still significantly lower compared to the stimulated control in the ADU-S100 and combination groups (p <0.001 ADU-S100; p <0.0001 combination) (**Figure 5I**). Perforin expression from CD8^+^ T cells was increased from 17% in the control to >30% in the ADU-S100 and combination treated cohorts (p <0.05), whereas stimulation had no effect (**Figure 5J**). IFN-γ expression from NK cells was significantly reduced in the spleens from 7.8% in the control to 3.9% in the ADU-S100 and combination cohorts (p <0.05). Stimulation significantly increased IFN-γ expression from NK cells in the cyto-IL-15 and ADU-S100 groups (p <0.05), and no differences we observed between the stimulated control and the stimulated treated groups (**Figure 5K**). In addition, there were no differences in perforin expression from NK cells and stimulation had no effect (**Figure S3E**, **Supplementary File 1**).

### STING activation with ADU-S100 leads to significant changes in gene expression and remarkable activation of immunological processes after six days of treatment initiation

**Figure 6:**
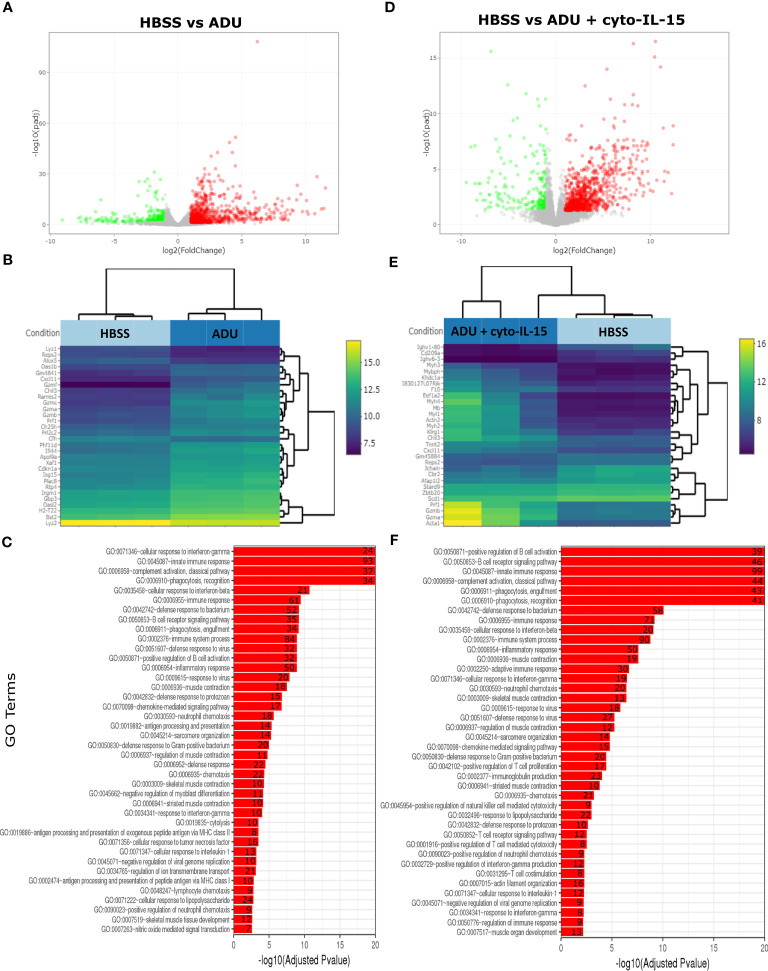
Treatment with ADU-S100 induces significant changes in gene expression in TRAMP-C2 prostate tumors. Differential gene expression analysis followed by gene ontology analysis between tumors treated with HBSS (control) versus ADU (left panel), and HBSS versus combination of ADU with cyto-IL-15 (right panel) (*n* = 3/cohort). **(A)** Volcano plots mapping the fold changes against adjusted p-values (padj) highlighting significantly differentially expressed genes. Upregulated significant genes are indicated by red dots (padj < 0.05 and log2 fold change >1), downregulated significant genes are green (padj < 0.05 and log2 fold change <-1), and non-significant genes are grey. **(B)** Bi-clustering heatmaps of the log2-transformed expression values in each sample showing the expression profiles of the top 30 differentially expressed genes. Blue colors indicate lower, while yellow colors indicate higher relative expression. **(C)** Gene ontology (GO) of the top 20 enriched functions ranked based on their log2-transformed p-value (< 0.05) for each of the comparisons. The size of a bubble represents the percentage of functional genes covered, while numbers next to the bars indicate the number of significantly DEG involved in each biological process.

The impact of ADU-S100 treatment on gene expression was investigated after 6 days of treatment in TRAMP-C2 tumors. RNA was isolated and RNA sequencing was performed in tumors treated with HBSS (control), cyto-IL-15, ADU-S100, or combination of cyto-IL-15 and ADU-S100 (*n* = 3 tumors per group). As shown in **Table 1**, when HBSS-treated tumors were compared with cyto-IL-15-treated tumors, a total of 96 genes were differentially expressed (**Figure S4**, **Supplementary File 1**). However, when the HBSS group was compared with ADU or combination, more than 1,000 genes were significantly differentially expressed, mainly upregulated (**Figures 6A, D**). When cyto-IL-15 treated tumors were compared with ADU-S100 or combination treated ones, 259 and 117 genes were differentially expressed (**Figures S4, S5**, **Supplementary File 1**). No major changes in differential gene expression were seen in the ADU-S100 versus combination comparison (12 DEGs) (**Figure S5**, **Supplementary File 1**). A list of all the differentially expressed genes in all comparison sets is provided in **Supplementary File 2** (SF2_DEG_analysis). **Figures 6B, E** show heatmaps of the top 30 differentially expressed genes in the HBSS versus ADU-S100 or versus combination comparisons, respectively. Heatmaps of DEGs in the rest of the comparisons are shown in **Figures S4, S5** (**Supplementary File 1**). Genes involved in chemokine (Cxcl11), granzyme (Gzma, Gzmb) and perforin (Prf1) activation were upregulated after treatment with ADU-S100 in both comparisons. Moreover, Ifi44 (Interferon-induced protein 44) and Irgm1 (Immunity Related GTPase M) genes were upregulated indicating exposure to IFN-γ. The Xaf1 (XIAP Associated Factor 1) gene was also upregulated. The protein of this gene inhibits the inhibitor of apoptosis (IAP) proteins, hence indicates initiation of apoptosis.

**Table 1:** Summary of significantly differentially expressed genes (DEG).

Comparison​	Upregulated genes​	Downregulated genes​	Total significantly DEGs
HBSS vs cyto-IL-15​	72	24	96
HBSS vs ADU​	733	307	1,040
HBSS vs combination	921	220	1,141
cyto-IL-15​ vs ADU	207	52	259
cyto-IL-15 vs combination	98	19	117
ADU vs combination	5	7	12

The gene ontology analysis showed a major immune activation with differentially expressed genes in both HBSS versus ADU-S100 and HBSS versus combination comparisons being involved in processes related to immune response, such as cellular response to IFN-γ and IFN-β, innate immune response, complement activation, immune and inflammatory responses, positive regulation of B cell activation, chemokine signaling pathway (**Figures 6C, F**). Positive regulation of NK-mediated cytotoxicity and T cell-mediated cytotoxicity were also among the top enriched functions in the HBSS versus combination comparison. Gene ontology analysis for the rest of the comparisons are shown in **Figures S4, S5** (**Supplementary File 1**). A list of all the differentially expressed genes involved in each GO function in all comparison sets is provided in **Supplementary File 3** (SF3_GO_analysis).

## Discussion

In the current study, the membrane-localizing cyto-IL-15 was combined with the STING agonist ADU-S100, and their efficacy was investigated in both syngeneic and humanized prostate cancer murine models. Our key findings indicate that the two agents acted synergistically to prolong mouse survival by delaying tumor growth or eliminating tumors in 58-67% of mice treated with the combination depending on tumor model. Moreover, treatment combination induced curative abscopal immunity in 50% of mice with bilateral tumors and offered long lasting immunity upon tumor rechallenge in 83% of mice previously cured by the combination treatment. The observed topical and systemic effects were due to a significantly enhanced immune response after combination treatment as measured in tumors, blood and spleens of treated mice, leading to induction of cell death.

Previous studies have demonstrated the impact of using IL-15 in combination immunotherapies for cancer. Combination of anti-PD-L1 antibodies with the IL-15 superagonist ALT-803 (N-803) led to additive effects in murine models of colon and breast cancer (31). In a phase I clinical trial, in patients with non-small cell lung cancer, combination of ALT-803 with nivolumab led to objective responses in 29% of the patients (32). Triple combination of IL-15 with antibodies to PD-L1 and CTLA-4 increased antitumor efficacy and prolonged survival of mice with colon and prostate tumors compared to monotherapies (33, 34). A phase I trial using rhIL-15 with nivolumab and ipilimumab is currently ongoing in patients with treatment-refractory and metastatic solid tumors (NCT03388632). Trials combining IL-15 with avelumab (anti-PD-L1) are also underway in patients with renal cell cancer and mature T-cell lymphoma (16). Our group has demonstrated the augmented efficacy of cyto-IL-15 compared with non-modified IL-15 used as monotherapy or in combination with membrane localizing inhibitory antibodies for CTLA-4 and PD-L1 (cyto-abs). Cyto-IL-15 alone or in combination with cyto-abs, administered intratumorally in subcutaneous prostate tumors in mice, delayed tumor growth and increased survival by expanding the infiltration of NK and CD8^+^ T cells in the tumors leading to tumor necrosis (12).

Since combination of checkpoint inhibitors with cyto-IL-15 did not lead to additional anti-tumor activity, we sought to investigate new combinations to improve the efficacy of cyto-IL-15. In an *in vitro* prostate cancer-lymphocyte co-culture model, we have shown that IL-15 combined with a STING agonist led to enhanced cancer cell killing due to activation of NK cells (23). In the present study, cyto-IL-15 combined with the STING agonist ADU-S100 led to significant anti-tumor efficacy and survival benefits in murine prostate cancer models. This combination regime was curative and offered both abscopal and long-lasting immunity to the majority of treated mice. The combination of STING agonists with anti-PD-1 therapy led to anti-tumor efficacy in a melanoma murine model (35). Furthermore, combination of ADU-S100 with PD-L1 and OX40 modulators enhanced clearance of breast tumors in mice (20). Ager et al. have shown that antibodies for PD-1, CTLA-4, and 4-1BB combined with a STING agonist can lead to bilateral tumor regression in 75% of mice with subcutaneous prostate tumors (36). Several STING agonists are currently in clinical trials, including ADU-S100, alone or in combination with checkpoint inhibitors (22). However, the clinical data have not been very promising so far in terms of survival benefit and disease regression. In a phase Ib trial, ADU-S100 combined with spartalizumab (anti-PD-1) led to partial responses only in PD-1–relapsed/refractory melanoma and PD-1-naïve triple negative breast cancer patients (37). Combination of STING agonists with cyto-IL-15 might be able to overcome this impediment without the need of combining three or four treatment agents, which could lead to increased toxicities.

To minimize systemic adverse effects, ADU-S100 and cyto-IL-15 were injected intratumorally. The membrane-localizing property of cyto-IL-15 required intratumoral injection for the agent to exert its potential in its entirety. However, being able to initiate not only local, but also abscopal immunity is crucial for intratumoral therapies. A previous study has shown that treatment with STING agonist monotherapy can lead to regression of uninjected melanoma tumors (38). Moreover, STING agonist combined with checkpoint modulation has been shown to elicit abscopal immunity against distal prostate tumors in mice (36). In the present study, ADU-S100 monotherapy also led to abscopal immunity but the effect was augmented with the combination. Our study is the first to our knowledge to demonstrate abscopal effects in mice with bilateral prostate tumors treated on a single site with a combination of a STING agonist (ADU-S100) and a cytokine (cyto-IL-15).

Intratumoral administration of cyto-IL-15 and ADU-S100 eliminated off-target toxicities. No weight loss was observed when these agents were used alone or in combination. However, intratumoral injection of STING agonist led to a skin ulceration at the injection site. Similar tissue pathology has been reported with other STING agonists/cyclic dinucleotides and the effect was found to be dependent on dose and tumor size (36). Adjusting and lowering the ADU-S100 dosage should be considered in the future to minimize such adverse effects. Lower doses of STING agonists might also be more beneficial as very high tumor-ablative doses can compromise T cell responses (38). Localized STING agonist administration have been previously shown to cause intratumoral hemorrhagic necrosis in a murine model of pancreatic cancer, which was associated with STING-mediated induction of TNF-α (39). Similarly, in our study significant production of TNF-α in tumors treated with ADU-S100 alone or in combination was reflected on the increased degree of intratumoral necrosis observed in the same tumors, with the amount of TNF-α being proportional to the extent of necrosis.

To investigate the mechanisms that drove the local and abscopal responses observed in the treated mice, cytokine induction was measured in blood plasma and in tumors. CCL2, CXCL10, IL-6, IFN-α and IFN-γ release was increased both in plasma and tumors of mice treated with the combination regime. However, activation was more profound inside the tumors with increases in CCL5, CXCL1, GM-CSF, IL-1β, IFN-β and TNF-α. ADU-S100 administered as monotherapy had a significant effect on release of CXCL1, IFN-β, IFN-γ, and TNF-α, but only intratumorally not systemically, which could explain the slightly reduced abscopal anti-tumor effects seen in mice treated with ADU-S100 alone. Induction of type I interferons was anticipated after treatment with the STING agonist and induction of IFN-γ could be due to the increase of NK and cytotoxic T cells after combination treatment as cyto-IL-15 can increase NK and CD8^+^ T cells proliferation and activation, whereas STING agonists can increase NK cell cytotoxicity and T cell priming by DC cells (11, 19). Moreover, the findings indicate that the presence of cyto-IL-15 augmented the capability of the STING agonist to induce type I interferons, especially IFN-α. Type I IFNs promote the DC stimulatory capacity towards CD8^+^ T cells and the migration of NK and CD8^+^ T cells towards tumors, throught induction of chemokines such as CXCL10 (40).

The combination of cyto-IL-15 with ADU-S100 increased the release of IFN-γ, which is in agreement with our previous *in vitro* findings (23). This could be due to the increased IL-15Rα expression in tumors treated with the combination regime. Since IL-15Rα expression was lower in tumors treated with cyto-IL-15 alone, we can assume that STING agonists, like ADU-100, augment the potential of IL-15 by increasing expression of the IL-15 receptor, IL-15Rα. This could also explain the synergistic anti-tumor effects observed in mice treated with the combination. Increasing the expression of IL-15Rα receptor could also increase transpresentation of IL-15, which is known to generate and maintain homeostasis of memory CD8^+^ T cells, hence leading to long-lasting T cell-mediated immunity (41).

To interrogate the systemic immune activation and understand what drove abscopal immunity and immunoprotection, the immune cell composition was measured in spleens from treated mice. Treatment with cyto-IL-15 and ADU-S100 combination led to an expansion of B cells, CD8^+^ T cells, NK and NKT cells. Moreover, it enhanced the proliferative and cytotoxic capabilities of T cells as indicated by increased Ki67^+^ and perforin^+^ CD8^+^ T cells. Upregulation of NK and CD8^+^ T cells is in line with the mechanism of action of IL-15. However, a decrease in the DCs, macrophages, CD4^+^ T cells, IFN-γ^+^ CD8^+^ T and IFN-γ^+^ NK cells populations was seen after treatment with ADU-S100 or combination. This could potentially be due to homing of these cells from the spleen to the tumor. A study in melanoma patients suggested that therapy-associated migration of antigen-reactive T cells from the periphery towards the tumor is associated with prolonged overall survival, and hence is crucial for successful therapy (42). To examine the immune composition inside the tumors, we prepared single cell suspensions from non-necrotic areas of TRAMP-C2 tumor collected from treated mice at day six after treatment. Suspensions were frozen (FBS with 10% DMSO) to be analyzed together; unfortunately, cells collected from mice treated with ADU-S100 or combination did not survive the freezing process, whilst cells from vehicle or cyto-IL-15 treated mice were alive. We hypothesized that most of the cells in tumors treated with ADU-S100 or combination were already in a late apoptotic phase when collected, and this combined with freezing resulted in their death. The increased degree of late apoptotic cells was evident in the histological analysis of these tumors as indicated by increased cleaved caspase-3 staining, a late apoptotic marker.

Hence, the tumor microenvironment was investigated using sequencing of RNA extracted from treated TRAMP-C2 tumors. Cyto-IL-15 as monotherapy led to a small number of genes being differentially expressed which might appear to be in contradiction to our previous findings (12). However, as shown previously, cyto-IL-15 exerts its anti-tumor effects approximately 14 days after treatment and samples in that study were collected at survival endpoint (~28 days post-treatment), whereas the tumors in the present study were collected only at 6 days post-treatment. RNA sequencing showed a very strong immune activation in tumors treated with ADU-S100 or combination, with upregulation of genes involved in expression of perforin and granzymes, which induce cytotoxicity and cell death mediated by cytotoxic T lymphocytes and NK cells (43). Moreover, the differentially expressed genes belonged to pathways regulating B cell activation, complement activation, innate and adaptive immune responses, chemokine signaling, T cell and NK cell mediated cytotoxicity and cellular responses to IFN-β and IFN-γ. Several previous studies have demonstrated that STING activation using agonists promotes priming and activation of T cells, tumor infiltration and cancer killing by T cells (44). A study has also shown that stimulation of STING by CDNs leads to autonomous activation of B cells in *in vitro* and *in vivo* models (45). In agreement with our findings, a study in preclinical mouse models has shown that induction of type I IFNs through STING activation, results in tumor rejection by enhancing NK cell activation and cytotoxicity either directly or by upregulating IL-15 and IL-15Rα receptors (46). A study in murine models of melanoma has also shown that while STING agonists can lead to regression of injected tumors, treatment of IL-15Rα−/− knockout mice with STING agonists had no effect on distant secondary tumors; hence, STING-mediated abscopal immunity required expression of IL-15 (47).

One of the limitations of our study was that due to the high degree of cell death in tumors treated was ADU-S100 (alone or in combination), it was not possible to isolate single cell suspensions from these samples to analyze the immune cell composition and activation, and measure antigen-specific T cell responses. Hence, in future studies lower doses of ADU-S100 should be used to reduce the amount of cell death and eliminate the uncreative pathology observed in the tumors. Moreover, it would be interesting to investigate whether the immunity observed with our treatment combination is tumor-specific or could lead to elimination of any tumor type, by using for example a bilateral model where the primary tumor is prostate, whereas the distal flank is injected with a different tumor. An orthotopic prostate cancer model, where tumor cells are injected directly into the prostate could also be used to represent primary tumors and allow for a better understanding of the prostate tumor microenvironment.

In conclusion, our study evaluated the combination of the membrane localizing cyto-IL-15 with the STING agonist ADU-S100 both administered intratumorally, in three different murine prostate cancer models. We have demonstrated that the two treatments acted synergistically in generating curative responses in the majority of treated animals and offered long-lasting immunity and protection against future tumors by generating effective topical and systemic immune responses. No toxicities were observed apart from the ulcerative pathology at the injection site caused by ADU-S100, which was resolved after approximately two weeks. Hence, this study highlights the curative potential of combining cyto-IL-15 with STING agonist immunotherapies in treating preclinical tumors. This combination regime, by activating both innate and adaptive immune responses, could provide great therapeutic benefit in patients with tumors of low immunogenicity, such as prostate cancer.

## Data availability statement

The datasets presented in this study can be found in online repositories. The names of the repository/repositories and accession number(s) can be found below: NCBI via accession ID GSE199704. (https://www.ncbi.nlm.nih.gov/geo/query/acc.cgi?acc=GSE199704).

## Ethics statement

The animal study was reviewed and approved by the Animal Welfare Ethical Review Body (AWERB) Committee of King’s College London, UK and by the Home Office, UK under Project Licence Number (PPL) P731DA7F1.

## Author contributions

EP designed and performed experiments, analyzed and interpreted data, wrote the manuscript, and obtained funding. AE performed experiments. PD obtained funding and contributed to concept design. CG created study, designed experiments, and obtained funding. All authors reviewed the manuscript and approved the final version.
