# Supplementary material for: Cyto-IL-15 synergizes with the STING agonist ADU-S100 to eliminate prostate tumors and confer durable immunity in mouse models
Source: Front Immunol. 2023 Jul 3;14:1196829. doi: 10.3389/fimmu.2023.1196829 (PMC10350564; doi:10.3389/fimmu.2023.1196829)
Supplement: Supplementary file 1 [file DataSheet_1.pdf]

Supplementary Material

Combination of the STING agonist ADU-S100 with cyto-IL-15 leads to complete tumor regression and immunoprotection against prostate tumors in *in vivo* mouse models

Efthymia Papaevangelou*, **Ana M. Esteves, Prokar Dasgupta, Christine Galustian***

*** Correspondence:** Efthymia Papaevangelou: epapaeva@sgul.ac.uk, Christine Galustian: christine.galustian@kcl.ac.uk

Supplementary Methods

RNA sequencing

RNA samples were quantified using Qubit 4.0 Fluorometer (Life Technologies, Carlsbad, CA, USA) and RNA integrity was checked with RNA Kit on Agilent 5300 Fragment Analyzer (Agilent Technologies, Palo Alto, CA, USA). RNA sequencing libraries were prepared using the NEBNext Ultra II RNA Library Prep Kit for Illumina following manufacturer's instructions (NEB, Ipswich, MA, USA). Briefly, mRNAs were first enriched with Oligo(dT) beads. Enriched mRNAs were fragmented for 15 minutes at 94 °C. First strand and second strand cDNAs were subsequently synthesized. cDNA fragments were end repaired and adenylated at 3'ends, and universal adapters were ligated to cDNA fragments, followed by index addition and library enrichment by limited-cycle PCR. Sequencing libraries were validated using NGS Kit on the Agilent 5300 Fragment Analyzer (Agilent Technologies, Palo Alto, CA, USA), and quantified by using Qubit 4.0 Fluorometer (Invitrogen, Carlsbad, CA). The sequencing libraries were multiplexed and loaded on the flowcell on the Illumina NovaSeq 6000 instrument according to manufacturer's instructions. The samples were sequenced using a 2x150 Pair-End (PE) configuration v1.5. Image analysis and base calling were conducted by the NovaSeq Control Software v1.7 on the NovaSeq instrument. Raw sequence data (.bcl files) generated from Illumina NovaSeq was converted into fastq files and de-multiplexed using Illumina bcl2fastq program version 2.20. One mismatch was allowed for index sequence identification.

After investigating the quality of the raw data, sequence reads were trimmed to remove possible adapter sequences and nucleotides with poor quality using Trimmomatic v.0.36. The trimmed reads were mapped to the Mus musculus reference genome GRCm38 using the STAR aligner v.2.5.2b. The STAR aligner uses an aligner that detects intron/exon junctions and helps align the entire read sequences. Unique gene hit counts were calculated by using featureCounts from the Subread package v.1.5.2. Only unique reads that fell within exon regions were counted. Since a strand-specific library preparation was performed, the reads were strand-specifically counted.

After extraction of gene hit counts, the gene hit counts table was used for downstream differential expression analysis. Using DESeq2, a comparison of gene expression between the groups of samples was performed. The Wald test was used to generate p-values and Log2 fold changes. Genes with adjusted p-values < 0.05 and absolute log2 fold changes > 1 were called as differentially expressed genes for each comparison. A gene ontology (GO) analysis was performed on the statistically

significant set of genes by implementing the software GeneSCF using Fisher exact test. The mgi GO list was used to cluster the set of genes based on their biological process and determine their statistical significance.

Supplementary Tables

Table S1. List of anti-mouse antibodies used for flow cytometry.

Target type	Target	Fluorophore	Clone	Source
	Dead cells	Zombie UV		Biolegend
Cell surface	Ki-67	Alexa Fluor (AF) 700	SolA15	ThermoFisher
Cell surface	CD45.2	BUV395	104	BD Biosciences
Cell surface	CD3	Brilliant Violet (BV) 786	17A2	BD Biosciences
Cell surface	CD4	BV711 CD4	GK1.5	Biolegend
Cell surface	CD8α	BV510 CD8	53-6.7	Biolegend
Cell surface	CD161(NK1.1)	APC-Cy7	PK136	Biolegend
Cell surface	CD11c	BUV737	N418	BD Biosciences
Cell surface	CD11b	PE-Cy7	M1/70	Biolegend
Cell surface	CD19	BV605	6D5	Biolegend
Cell surface	F4/80	PerCP-Cy5.5	BM8	Biolegend
Cell surface	CD38	PE-Dazzle 594	90	Biolegend
Intracellular	FoxP3	BV421	MF23	Biolegend
Intracellular	Perforin	APC	S16009A	Biolegend
Intracellular	IFN-γ	BV 650	XMG1.2	Biolegend

Table S2. Gating strategy for unique immune cell subsets.

Cell population	Gating strategy
$CD8^+$ T cells	Live cells/ Singlets/ $CD45.2^+$/ $CD3^+$ / $CD8\alpha^+$
$CD4^+$ T cells	Live cells/ Singlets/ $CD45.2^+$/ $CD3^+$/ $CD4^+$ /$FoxP3^-$
$CD4^+$ T_{reg} cells	Live cells/ Singlets/ $CD45.2^+$/ $CD3^+$ / $CD4^+$ /$FoxP3^+$
NK cells	Live cells/ Singlets / $CD45.2^+$ /$CD3^-$ / $NK1.1^+$
NKT cells	Live cells/ Singlets/ $CD45.2^+$ /$CD3^+$ $NK1.1^+$
B cells	Live cells/ Singlets/ $CD45.2^+$ / $CD3^-$/ $CD19^+$
Dendritic cells (DCs)	Live cells/ Singlets/ $CD45.2^+$ / $CD3^-$/ $CD11c^+$ /$CD11b^-$
Myeloid derived suppressor cells (MDSCs)	Live cells/ Singlets/ $CD45.2^+$ / $CD3^-$/ $CD11b^+$ /$F4/80^-$
Macrophages	Live cells/ Singlets/ $CD45.2^+$ / $CD3^-$ $CD11b^+$ $F4/80^+$
M1-like macrophages	Live cells/ Singlets/ $CD45.2^+$ / $CD3^-$ / $CD11b^+$ / $F4/80^+$/ $CD38^+$
M2-like macrophages	Live cells/ Singlets/ $CD45.2^+$ / $CD3^-$ / $CD11b^+$ / $F4/80^+$/ $CD38^-$

Table S3. Tumor growth inhibition rate and combination index of TRAMP-C1 and TRAMP-C2 tumors in mice treated with HBSS (vehicle), cyto-IL-15, ADU-S100 or combination of cyto-IL-15 with ADU-S100.

Treatment	Mean tumor volume	Tumor growth inhibition rate (TGIR)[1]	Expected tumor growth inhibition rate (ETGIR)[2]	Combination index (CI)[3]
TRAMP-C1	**Day 20 post-treatment**			
**HBSS (vehicle)**	771.5			
**Cyto-IL-15**	284.3	0.631		
**ADU-S100**	120.8	0.843		
**Combination: cyto-IL-15 + ADU-S100**	13.67	0.982	0.942	**0.307**
TRAMP-C2	**Day 20 post-treatment**			
**HBSS (vehicle)**	788.3			
**Cyto-IL-15**	434.3	0.449		
**ADU-S100**	264.0	0.665		
**Combination: cyto-IL-15 + ADU-S100**	21.17	0.973	0.815	**0.146**

[1]Mean growth inhibition rate: 1 − (mean tumor volume of treated group at day 20 / mean tumor volume of vehicle group at day 20).

[2]Expected growth inhibition rate: $(TGIR_{\text{cyto-IL-15}} + TGIR_{\text{ADU-S100}}) - (TGIR_{\text{cyto-IL-15}} \times TGIR_{\text{ADU-S100}})$.

[3]CI: $(1 - TGIR_{\text{combination}}) / (1 - ETGIR)$

The CI value defines synergism (CI < 1), addition (CI = 1) and antagonism (CI > 1).

Supplementary Figures

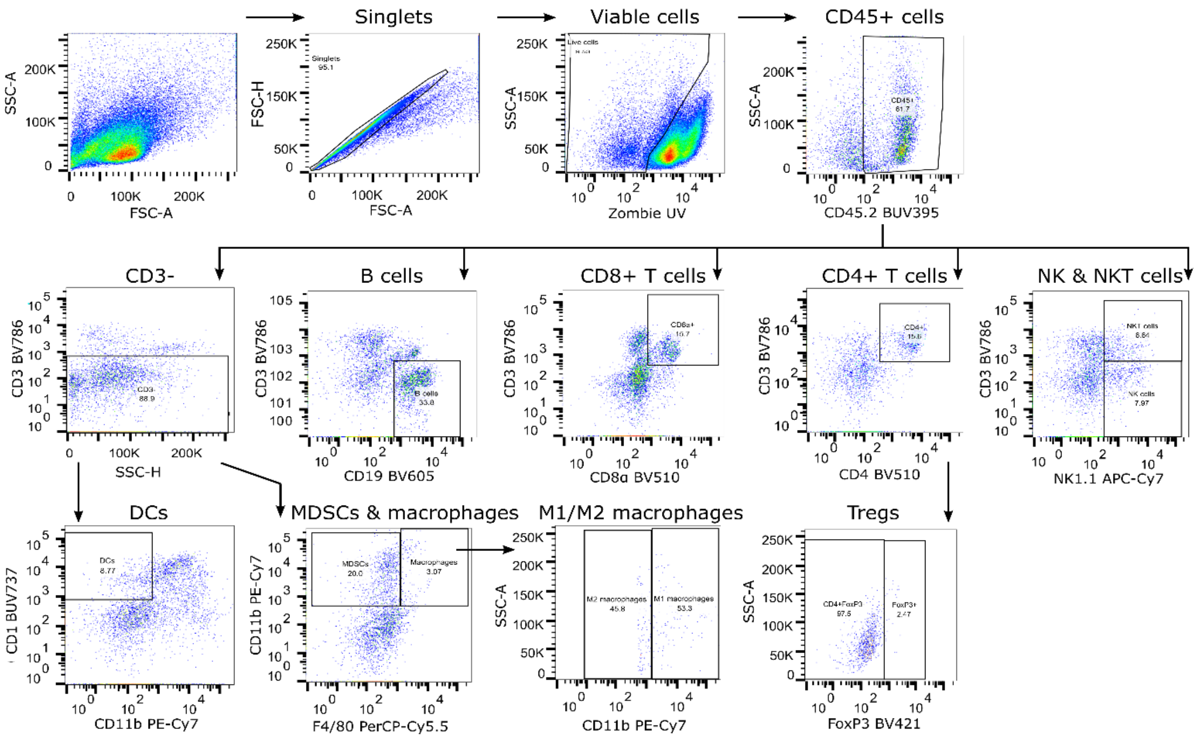

Figure S1. Representative gating strategy illustrating splenocytes being subgated to different unique immune cell populations.

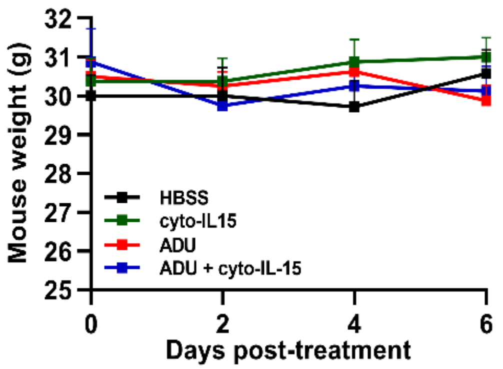

Figure S2. Effects of ADU-S100 and cyto-IL-15 combination on mouse weight. Mice with TRAMP-C2 tumors treated with HBSS, cyto-IL-15, ADU, or ADU and cyto-IL-15 combination were weighted every other day for the first week of treatment. Mice weights from all mice per group are shown.

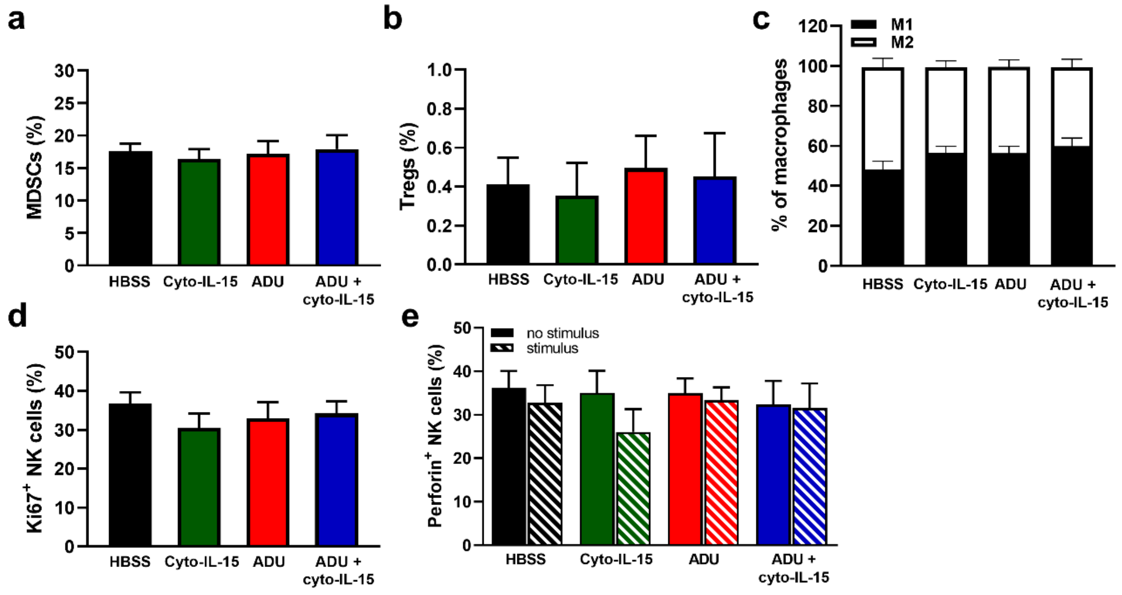

Figure S3. Analysis of immune cell composition of splenocytes in treated mice with TRAMP-C2 prostate tumors. Mice with TRAMP-C2 tumors (~200mm^3) were treated intratumorally with HBSS, cyto-IL-15, ADU or combination of ADU and cyto-IL-15 and splenocytes were harvested after 6 days of treatment initiation to be analysed using flow cytometry. **(a, b)** Frequencies of **(a)** MDSCs and **(b)** regulatory T cells (Tregs) subsets within the CD45$^+$ immune cell population. **(c)** Frequencies of M1 and M2 within the macrophages subset. **(d, e)** Frequencies of **(d)** Ki67$^+$ and **(e)** perforin$^+$ NK cells of splenocytes with or without PMA and ionomycin stimulation for 4 h. Results are means +1 SEM of measurements made for *n* = 6 mice per cohort compared using one-way ANOVA with Dunnett's multiple comparisons post-test.

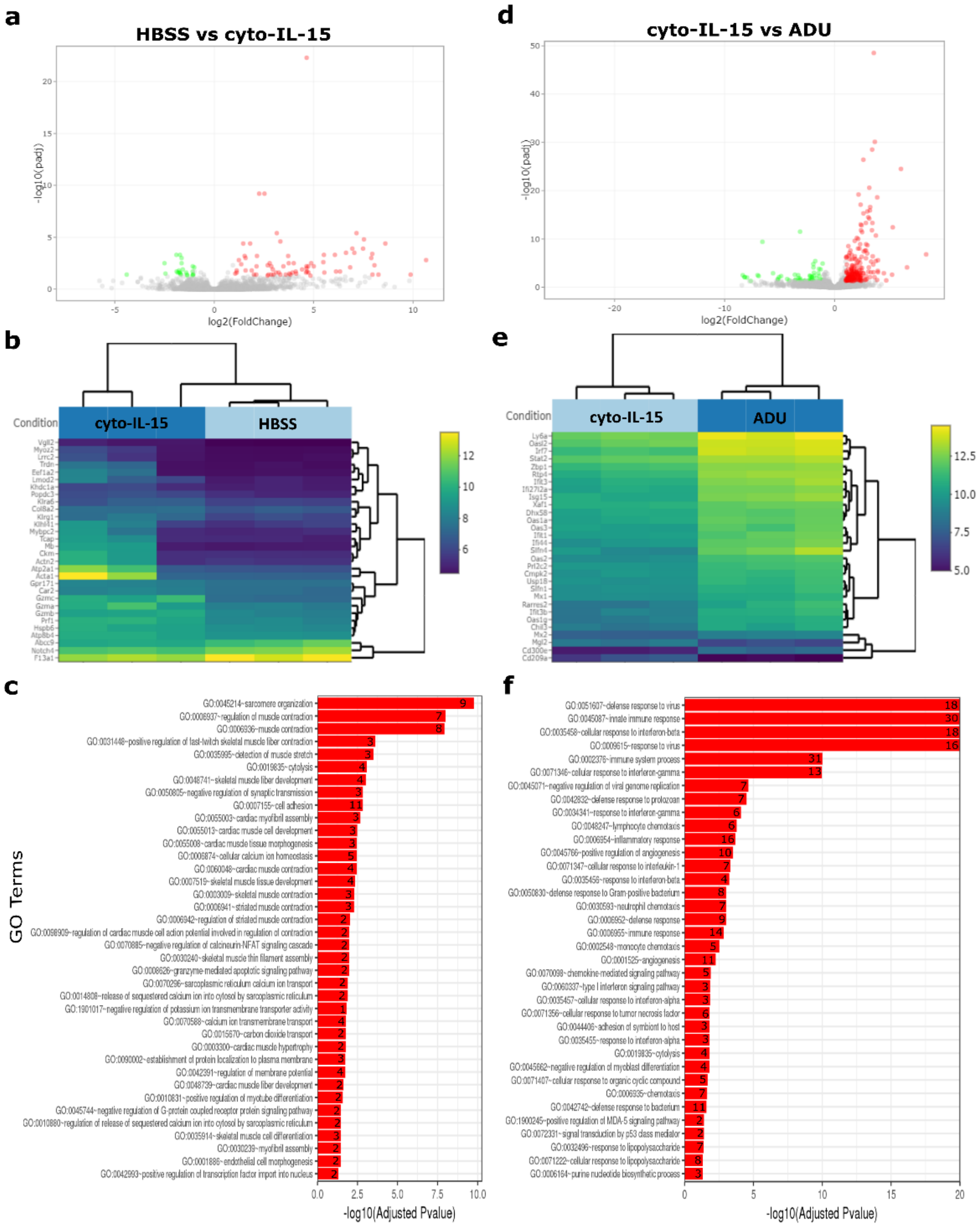

Figure S4. Effects of intratumoral treatment in gene expression in TRAMP-C2 prostate tumors. Differential gene expression analysis followed by gene ontology analysis between tumors treated with HBSS (control) versus cyto-IL-15 (left panel), and cyto-IL-15 versus ADU (right panel) (n = 3/ cohort). (a) Volcano plots mapping the fold changes against adjusted p-values (padj) highlighting significantly differentially expressed genes. Upregulated significant genes are indicated by red dots (padj < 0.05 and log2 fold change >1), downregulated significant genes are green (padj < 0.05 and log2 fold change <-1), and non-significant genes are grey. (b) Bi-clustering heatmaps of the log2-transformed expression values in each sample showing the expression profiles of the top 30 differentially expressed genes. Blue colours indicate lower, while yellow colours indicate higher relative expression. (c) Gene ontology (GO) of the top 20 enriched functions ranked based on their log2-transformed p-value (< 0.05) for each of the comparisons. The size of a bubble represents the percentage of functional genes covered, while numbers next to the bars indicate the number of significantly DEG involved in each biological process.

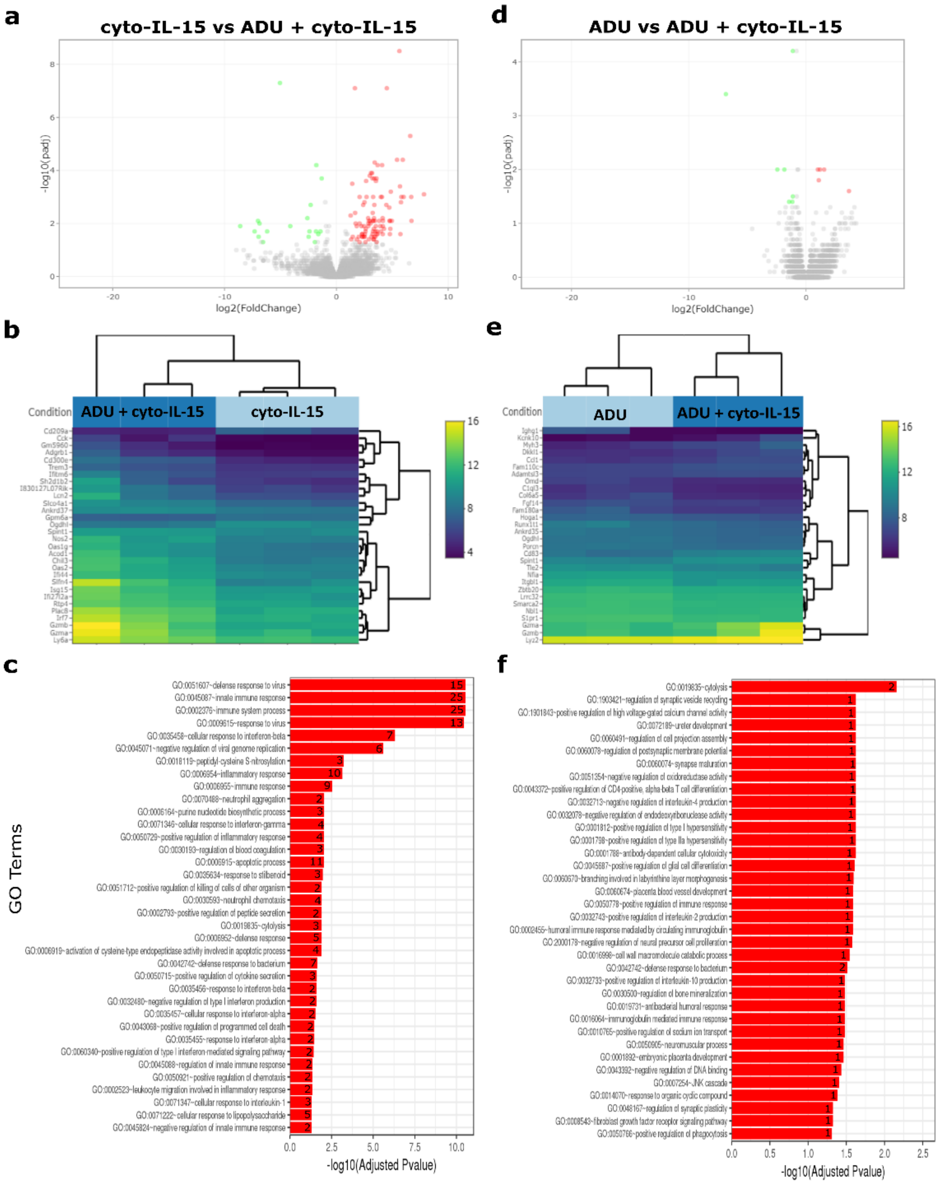

Figure S5. Effects of intratumoral treatment in gene expression in TRAMP-C2 prostate tumors. Differential gene expression analysis followed by gene ontology analysis between tumors treated with cyto-IL-15 versus combination of ADU with cyto-IL-15 (left panel), and ADU versus combination of ADU with cyto-IL-15 (right panel) (n = 3/ cohort). (a) Volcano plots mapping the fold changes against adjusted p-values (padj) highlighting significantly differentially expressed genes. Upregulated significant genes are indicated by red dots (padj < 0.05 and log2 fold change >1), downregulated significant genes are green (padj < 0.05 and log2 fold change <-1), and non-significant genes are grey. (b) Bi-clustering heatmaps of the log2-transformed expression values in each sample showing the expression profiles of the top 30 differentially expressed genes. Blue colours indicate lower, while yellow colours indicate higher relative expression. (c) Gene ontology (GO) of the top 20 enriched functions ranked based on their log2-transformed p-value (< 0.05) for each of the comparisons. The size of a bubble represents the percentage of functional genes covered, while numbers next to the bars indicate the number of significantly DEG involved in each biological process.
